# SMALL: open surgery versus minimally invasive vacuum-assisted excision for small screen-detected breast cancer—protocol for a phase III randomised multicentre trial

**DOI:** 10.1136/bmjopen-2025-099702

**Published:** 2025-04-08

**Authors:** Kenneth Elder, Charlotte Coles, David Dodwell, Beatrix Elsberger, Jessica Foster, Claire Gaunt, Julia R Henderson, Iain Lyburn, Claire Mabena, Jenna Morgan, Zohal Nabi, Sangeetha Paramasivan, Sarah Pinder, Sarah Pirrie, Shelley Potter, Tracy Roberts, Nisha Sharma, Elizabeth Southgate, Hilary Stobart, Amruta Talwalkar, Sian Taylor-Phillips, William Teh, Elliot Turner, Matthew G Wallis, Dan Rea, Stuart McIntosh

**Affiliations:** 1 Breast Unit, Western General Hospital, Edinburgh, UK; 2 Department of Oncology, University of Cambridge, Cambridge, UK; 3 Population Health, University of Oxford, Oxford, UK; 4 Aberdeen Royal Infirmary, Aberdeen, UK; 5 Cancer Research UK Clinical Trials Unit, University of Birmingham, Birmingham, UK; 6 Linda McCartney Centre, Royal Liverpool University Hospital, Liverpool, UK; 7 Gloucestershire Hospitals NHS Foundation Trust, Cheltenham, UK; 8 Medical Imaging Centre Cobalt Medical Charity, Cheltenham, UK; 9 Cranfield University, Cranfield, Wiltshire, UK; 10 Central and East London Breast Screening Services, Royal Free London NHS Foundation Trust, London, England, UK; 11 Division of Clinical Medicine, The University of Sheffield Medical School, Sheffield, UK; 12 National Radiotherapy Trials QA Group, Mount Vernon Cancer Centre, Northwood, England, UK; 13 Population Health Sciences, Bristol Medical School, University of Bristol, Bristol, England, UK; 14 School of Cancer and Pharmaceutical Sciences, King’s College London, London, England, UK; 15 Cancer Research Clinical Trials Unit, University of Birmingham, Birmingham, UK; 16 Translational Health Sciences, Bristol Medical School, University of Bristol, Bristol, England, UK; 17 Bristol Breast Care Centre, Southmead Hospital, Bristol, England, UK; 18 Institute of Applied Health Research, College of Medical and Dental Sciences, University of Birmingham, Birmingham, England, UK; 19 Breast Unit, St James’s Hospital, Leeds Teaching Hospitals NHS Trust, Leeds, Leeds, UK; 20 Independent Cancer Patients’ Voice, London, UK; 21 Wrightington Wigan and Leigh NHS Foundation Trust, Wigan, UK; 22 Warwick Medical School, University of Warwick, Coventry, England, UK; 23 Central and East London Breast Screening Service, Royal Free London NHS Foundation Trust, London, England, UK; 24 Cambridge Breast Unit, Cambridge University Hospitals NHS Foundation Trust, Cambridge, England, UK; 25 NIHR Cambridge Biomedical Research Centre, Cambridge University Hospitals NHS Foundation Trust, Cambridge, England, UK; 26 Patrick G Johnston Centre for Cancer Research, Queen’s University Belfast, Belfast, Northern Ireland, UK

**Keywords:** Breast surgery, Breast imaging, Clinical Trial, Breast tumours

## Abstract

**Introduction:**

Mammographic screening identifies many women with small breast cancers with favourable biological features, which have an excellent prognosis. Some of these may never have become clinically apparent without screening and are commonly described as ‘overdiagnosed’ cancers. Despite this, all patients with screen-detected cancers are currently treated with surgical excision and sentinel lymph node biopsy, although this may represent overtreatment. There is, therefore, a need for less invasive approaches to reduce treatment burden for patients while maintaining current excellent oncological outcomes. Vacuum-assisted excision (VAE) may represent such an alternative treatment approach, and the SMALL (**Open Surgery versus Minimally invasive-vacuum Assisted excision for smaLL screen-detected breast cancer**) trial aims to investigate the use of VAE for the safe de-escalation of surgical treatment for such excellent prognosis invasive breast cancers.

**Methods:**

SMALL is a prospective, multicentre, randomised phase III trial of VAE versus surgery in patients with small, biologically favourable screen-detected invasive breast cancer. SMALL has an innovative hybrid design with coprimary endpoints. These include a randomised non-inferiority comparison of surgical re-excision rates following initial treatment, and a single-arm analysis of local recurrence at 5 years following VAE. Secondary outcomes include complication rates, overall survival, quality of life and a health economic analysis. The trial includes a QuinteT Recruitment Intervention to support recruitment.

**Ethics and dissemination:**

Ethical approval was obtained from the Office for Research Ethics (Northern Ireland) for all UK sites. Results will be submitted for publication in a peer-reviewed journal, presented, shared with patient partners and with relevant professional organisations to inform future guideline development for the management of screen-detected breast cancer.

**Trial registration number:**

ISRCTN12240119.

**STRENGTHS AND LIMITATIONS OF THIS STUDY**

- Large multicentre randomised trial evaluating minimally invasive treatment for good prognosis screen-detected invasive breast cancer in comparison with standard surgery.
- Innovative hybrid 2:1 randomised design is efficient, maximising data from vacuum-assisted excision arm while limiting selection bias and provides embedded controls for local recurrence outcome.
- Patient and public involvement at all stages of the study design, funding and delivery.
- Radiotherapy and endocrine therapy are mandated in this trial, although some patients may prefer to de-escalate these treatment modalities in preference to surgery.

## Introduction

 Breast cancer is the most common cancer in women globally and accounts for 11.6% of all malignancies, with an estimated 2.1 million new cases and 627 000 deaths from the disease in 2018.^1^ The incidence of breast cancer has increased in recent decades, with mammographic screening programmes contributing to this rise.^2^,^4^ Historically, randomised trials of mammographic screening have estimated reductions in breast cancer-specific mortality in invited women of between 0% and 28%.^5 6^ However, there has been extensive debate around the benefits and harms of breast screening. In the UK, an Independent Breast Screening Review concluded that the relative risk of breast cancer mortality for women invited for screening compared with controls was 0.8, corresponding to a 20% reduction in breast cancer mortality.^7^,^9^ However, this review also noted that mammographic breast screening results in overdiagnosis—the detection of breast cancers which would never have become clinically apparent had a woman not attended for screening. Recent data confirm that small invasive breast cancers with favourable biological features have an excellent prognosis, and that such cancers may account for a significant proportion of screening overdiagnoses.^10^ The screening review estimated that for every breast cancer death prevented by screening, three women will be overdiagnosed and consequently overtreated and highlighted a need for less invasive treatment of screen-detected disease.^9^

To date, however, all patients with screen-detected breast cancers have been treated with surgery. This approach has remained unchanged since screening began, having been extrapolated from women with symptomatic disease rather than based on prospective evidence from a screened population. In the UK, 90% of patients with screen-detected breast cancers ≤15 mm in maximum diameter undergo breast-conserving surgery, breast irradiation and axillary sentinel node biopsy.^11^ Such treatment has an associated rate of complications, including poor cosmetic outcomes, which are known to be associated with reduced quality of life and with psychosocial morbidity.^12^,^14^

Taken together, overdiagnosis within screening programmes coupled with the morbidities of standard treatment mean that there is a need to identify less invasive treatment strategies for good prognosis disease to reduce treatment burden while maintaining good oncological outcomes. Although a number of minimally invasive treatment strategies have been described with promising results, ablative technologies, such as cryoablation or radiofrequency ablation, disrupt tumour tissue, leaving no specimen for histopathological assessment.^15^ Furthermore, most studies are small cohort studies with a lack of randomised evidence to support changes in practice.^16 17^ Vacuum-assisted biopsy (VAB) is a widely available minimally invasive technique which uses a large calibre needle to sample lesions using image guidance (ultrasound or mammographic) under local anaesthesia. Initially used for diagnosis, VAB has evolved and now has an evidence base for the treatment of benign lesions as well as the management of lesions of uncertain malignant potential (B3 lesions).^18^,^20^ This suggests that postprocedure imaging can accurately estimate complete removal of lesions in 90% of cases, with many women consequently able to avoid surgery.^21^,^23^ Furthermore, the procedure has been shown to be well tolerated by patients.^24^ There is sufficient evidence to support the repurposing of vacuum-assisted biopsy (VAE) for the minimally invasive treatment of small screen-detected breast cancers with biologically favourable characteristics, although prospective randomised evidence will be required to underpin the introduction of this technique into routine clinical practice.

The SMALL trial aims to generate high-quality prospective randomised evidence for the de-escalation of surgical treatment and evaluate minimally invasive VAE as an alternative to standard surgery for small, biologically favourable, screen-detected invasive breast cancers.

## Methods

### Study design

SMALL is a prospective, multicentre, randomised phase III trial of minimally invasive VAE versus surgery in patients with small, biologically favourable screen-detected invasive breast cancer. It aims to generate high-quality, practice-changing clinical evidence to support the safe de-escalation of surgical treatment in conjunction with standard adjuvant radiotherapy and endocrine therapy in selected patients. The study is designed to assess whether:

- The extent of surgical treatment can be reduced alongside standard adjuvant radiotherapy and endocrine therapy.
- Vacuum-assisted excision (VAE) is non-inferior to conventional surgery in terms of the requirement for a second operation to achieve complete resection of the cancer.
- There is an acceptable local recurrence risk in the VAE arm with long-term follow-up.
- Sentinel node biopsy can be safely omitted in low-risk patients undergoing VAE.

As event rates in early breast cancer are low, with local recurrence rates in the region of 1% at 5 years,^25^ a randomised non-inferiority trial comparing local recurrence rates between VAE and standard surgery was considered to be unfeasible, as it would require large patient numbers, a lengthy recruitment period and long follow-up. A hybrid design was therefore adopted, with two coprimary endpoints.

The first coprimary endpoint is a non-inferiority comparison of postprocedure surgical re-excision rates, defined as the number of patients who require a second procedure to ensure complete removal of their cancer following either VAE or surgery. Patients will be randomised in a 2:1 ratio in favour of VAE, with randomisation required for this comparison in light of the inherent variation associated with surgical interventions.

The second coprimary endpoint is a single-arm analysis of local recurrence rates at 5 years following VAE, with a predetermined ‘unacceptable’ level of local recurrence set at 3% at 5 years in conjunction with patients and clinicians.

The 2:1 randomisation increases the likelihood of patients avoiding standard surgery and means that maximal data on VAE can be collected. The embedded controls in the standard surgery arm not only will reduce the risk of selection bias seen in single-arm studies but also will provide a contemporaneous group of patients undergoing standard treatment, which will aid interpretation of the local recurrence data in the single-arm analysis.

### Study setting

SMALL is a hospital-based trial, which will open in up to 70 tertiary care breast units across the United Kingdom (see Participating Hospitals, below).

### Study duration

The first SMALL recruiting site opened in December 2019. The current projected end of recruitment date is 30 June 2026.

### Eligibility criteria

Eligible patients are aged over 47 years, with unifocal screen-detected invasive breast cancer with a maximum tumour size of 15 mm on imaging. Tumours should have favourable biological features on diagnostic core biopsy, defined as grade 1 disease, which is strongly oestrogen and progesterone receptor positive and HER2 negative, and have negative axillary staging at diagnosis. Full inclusion/exclusion criteria are summarised in table 1. The participant information sheet and informed consent form can be found in the online supplemental materials.

**Table 1:** SMALL trial inclusion/exclusion criteria

Inclusion criteria	Exclusion criteria
Screen detected breast cancer	Associated malignant microcalcification outwith the lesion
Age over 47 years	Bilateral breast cancer
Radiological unifocal disease	Invasive lobular cancer
Maximum tumour size of 15 mm on mammography/ultrasound	Not strongly ER/PR positive or HER2 positive
Grade 1 tumour	Inability to provide informed consent
Strongly ER/PR positive disease (Allred score≥7 or >66% staining)	Unable or unwilling to undergo standard surgical treatment
HER2 negative (0/1+on immunohistochemistry or 2+ with negative in situ hybridisation).	Contra-indications to standard adjuvant therapies (radiotherapy or endocrine therapy)
Normal axillary US or radiologically equivocal axillary ultrasound with benign pathology on subsequent FNA or core biopsy	Previous ipsilateral invasive breast cancer or DCIS
No previous diagnosis of ipsilateral breast cancer (in situ or invasive)—contralateral disease permitted if surgically treated>5 years previously and disease free	Other invasive malignancy unless Disease free for≥5 years or Previous basal cell carcinoma, cervical carcinoma in situ, superficial bladder cancer

SMALL - **Open Surgery versus minimally invasive-vacuum assisted excision for small screen-detected breast cancer**

DCISductal carcinoma in situERoestrogen receptorFNAfine needle aspirationHER2human epidermal growth factor receptor type 2PRprogesterone receptorUSultrasound

### Interventions and patient pathways

**Figure 1:**
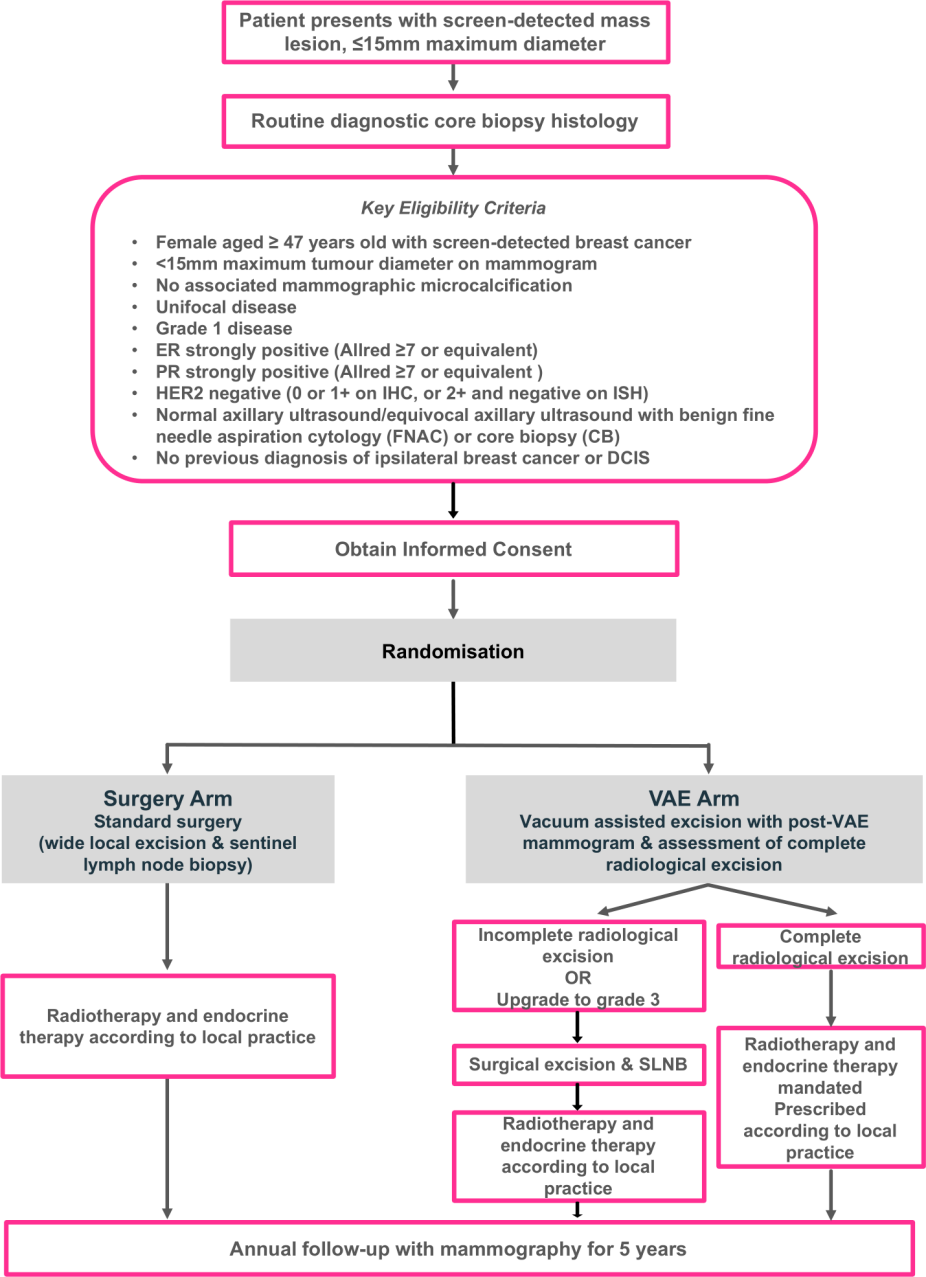
SMALL (**Open Surgery versus minimally invasive-vacuum assisted excision for small screen-detected breast cancer**) trial schema showing key eligibility criteria. DCIS, ductal carcinoma in situ; ER, oestrogen receptor; HER2, human epidermal growth factor receptor type 2; PR, progesterone receptor; VAE, vacuum-assisted excision; ISH, in situ hybridisation; IHC, immunohistochemistry; SLNB, sentinel lymph node biopsy.

The trial schema is shown in figure 1. Participants are randomised to receive standard surgical treatment or VAE of the tumour under local anaesthesia.

Patients randomised to surgery will undergo standard surgical treatment under general anaesthesia (including sentinel lymph node biopsy (SLNB) with surgical re-excision of involved margins as deemed necessary by local protocol). Adjuvant endocrine therapy and radiotherapy are not mandated in surgery arm patients and should be given according to local protocol. This may include omission of these therapies if this is standard local practice in the management of low-risk invasive breast cancer.

Patients randomised to VAE will undergo the procedure under local anaesthetic, with insertion of a marker clip at the tumour site. A post-VAE mammogram will be performed to check the marker clip position. The completeness of excision will be determined radiologically, based both on the operator’s impression of complete excision during the VAE procedure and the postoperative mammographic appearances. SLNB will not be performed in the VAE arm of the study where excision is determined to be complete, patients will proceed to protocol-mandated adjuvant radiotherapy and endocrine therapy. These may be prescribed according to local protocols and may include partial breast radiotherapy. A radiotherapy quality assurance programme is implemented by the National Radiotherapy Trials QA Group to ensure the safety and consistency of radiotherapy delivery at participating sites.

If the lesion is deemed to be incompletely excised, then the patient should undergo surgical re-excision (as per the standard surgery arm of the trial). Histopathological examination of excised tissue will be carried out following VAE. Although it will not be possible to confirm complete excision pathologically, this will confirm the grade of the excised lesion. Cancers upgraded to grade 2 following VAE may remain in the study due to the similar biological behaviour of these lesions to grade 1 cancer. However, where pathologists report an upgrade to grade 3 disease, patients should undergo standard surgery due to the biologically more aggressive nature of the disease and greater probability of nodal metastases.

All patients will be followed up with 5 years of annual mammography, with long-term follow-up data being obtained by linkage to routinely collected National Health Service data.

### Outcomes

#### Primary outcome measures

As outlined above, SMALL has coprimary endpoints.

#### Re-excision following primary procedure

Existing data show that the re-excision rate following standard breast-conserving surgery for screen-detected breast cancer is consistently 15%–20%.^11^ After consultation with patient advocates during study development, it was determined that a second procedure rate following VAE of up to 10% higher than that following surgery would be deemed acceptable. A non-inferiority margin of 10% was therefore set for this randomised comparison.

#### Local recurrence following VAE

The risk of breast cancer local recurrence is known to be around 1% at 5 years following standard wide local excision with adjuvant therapies, and that local recurrence does not impact survival.^25^ It is possible that local recurrence risk may be increased following VAE due to incomplete resection as assessed radiologically rather than histopathologically. However, what is critical to long-term clinical outcomes is the significance of any residual low-volume disease and its impact on local recurrence and survival. Even in cases where additional surgery is not carried out for focally involved resection margins, acceptable local recurrence rates (<3%) at 5 years can be obtained with the use of adjuvant endocrine therapy and radiotherapy.^26^

An acceptable level of local recurrence was discussed with patient advocates during trial development. It was determined that a local recurrence rate of ≥3% at 5 years would be deemed unacceptable to patients undergoing VAE, with the recognition that local recurrence is not a life-threatening event and may be salvaged with additional surgical treatment. Therefore, a single-arm intention-to-treat analysis of local recurrence rates will be carried out, with reference to both the predefined unacceptable level of 3% and the local recurrence rate within the surgery arm of the study.

### Secondary outcomes

Secondary outcome measures in SMALL are as follows:

1. Protocol-defined complications arising within 30 days of surgery or VAE.
2. Time to ipsilateral breast cancer recurrence.
3. Time to development of contralateral breast cancer
4. Overall survival
5. Quality of life—this outcome will examine the hypothesis that the psychological well-being of women undergoing minimally invasive VAE of small screen-detected breast cancers is not adversely affected by this approach, when compared with standard surgical treatment. Assessment will use the EORTC QLQ-C30 and BR23, the EuroQoL EQ-5D and the breast-conserving therapy module of the BREAST-Q.
6. Quality-adjusted life year (QALY)—calculated from the EQ-5D QoL questionnaire.

### Sample size calculation

The total number of patients to be recruited with a 2:1 randomisation ratio is 800 (533 VAE, 267 surgery). The total number required for the surgical re-excision comparison is 762 patients, and this has been inflated by 5% to ensure sufficient patients for the single-arm analysis of local recurrence rates following VAE and to allow for possible dropouts. To ensure that the trial as a whole only has 5% alpha, the significance level for each coprimary outcome has been set at 2.5% with 90% power. The probability of success in both the surgery arm and the VAE arm is expected to be 80% (20% re-excision). The maximal acceptable difference between the two has been set at 10%, which was defined as acceptable by our patient partners bearing in mind that this is salvageable by a second procedure and has no survival sequelae. The total number of patients required for the local recurrence-free survival outcome, analysed on an intention to treat basis is 511.^27^

### Health economic outcomes

If VAE is found to be an effective approach for the treatment of good prognosis screen-detected early invasive breast cancer, then it is likely that there will be important cost benefits for the healthcare sector. An economic evaluation will be carried out to determine the cost-effectiveness of VAE compared with surgery in this setting. A cost-effectiveness analysis will be undertaken based on a number of outcomes including the cost re-excision rate avoided at 5 years and cost per local recurrence of breast cancer avoided utilising the clinical outcome data collected within the trial. In addition, a cost-utility analysis will be undertaken to calculate the cost per additional QALY gained.

### Mammographic image library

SMALL will generate a library of deidentified mammographic images, with the aim being for future studies to identify potential radiological features that could determine cases where minimally invasive treatment was associated with early local recurrence.

### QuinteT Recruitment Intervention

SMALL will employ an integrated QuinteT Recruitment Intervention (QRI) aimed at optimising recruitment and informed consent.^28^ The QRI uses a novel qualitative and mixed methods approach pioneered during the Health Technology Assessment-funded ProtecT study and has been shown to support recruitment to time and target in other challenging randomised trials.^29^ The QRI will have two iterative phases. Phase I will aim to understand recruitment processes, using a combination of mapping of recruitment pathways, audio-recording of recruitment discussions and in-depth interviews with recruiters and patients. Phase II will use the findings from phase I to develop interventions to support and improve recruitment, including ‘recruitment tips’ documents, recruitment workshops and the provision of centre-specific feedback.

### Patient and public involvement

Patients have been involved with SMALL since the study’s inception and were involved in consultations around the design of the study and its acceptability to patients. The non-inferiority margin and the unacceptable local recurrence threshold were set in conjunction with patient advocates. Two patient advocates were co-applicants on the SMALL trial funding application, and there is a patient advocate on the Trial Management Group. All patient-facing documents have been developed and revised in conjunction with patient advocates.

### Monitoring

On-site monitoring will be carried out as documented in the trial Quality Management Plan. Central monitoring will be carried out by regular scrutiny of Case Report Forms for protocol compliance, data completeness and consistency.

The trial Data Monitoring Committee (DMC) will scrutinise trial data, including recruitment, conduct, data completeness, compliance, safety and complications. The DMC will also monitor local recurrence events to ensure that these do not exceed a pre-determined unacceptable threshold of 3% per annum, set in close consultation with patient and public involvement members of the trial development group.

### Ethics and dissemination

The study has been approved by the Health and Social Care Research Ethics Committee Northern Ireland (reference 19/NI/0126). Informed written consent will be obtained from all participants before taking part in the trial. Data will be available on reasonable request to the chief investigator on completion of the trial and after publication of the results.

Study results will be published in a peer-reviewed journal and presented at relevant specialty conferences. Findings will be shared with the relevant professional organisations to inform future guideline development where appropriate.
