# Supplementary material for: SMALL: open surgery versus minimally invasive vacuum-assisted excision for small screen-detected breast cancer—protocol for a phase III randomised multicentre trial
Source: BMJ Open. 2025 Apr 8;15(4):e099702. doi: 10.1136/bmjopen-2025-099702 (PMC11979497; doi:10.1136/bmjopen-2025-099702)
Supplement: Supplementary file 1 [file bmjopen-2025-099702.reviewer_comments.pdf]

PEER REVIEW HISTORY

BMJ Open publishes all reviews undertaken for accepted manuscripts. Reviewers are asked to complete a checklist review form and are provided with free text boxes to elaborate on their assessment. These free text comments are reproduced below.

ARTICLE DETAILS

Title (Provisional)

SMALL: Open Surgery versus minimally invasive-vacuum assisted excision for small screen-detected breast cancer – protocol for a phase III randomised multicentre trial

Authors

Elder, Kenneth; Coles , Charlotte; Dodwell, David; Elsberger, Beatrix; Foster, Jessica; Gaunt, Claire; Henderson, Julia R; Lyburn, Iain; Mabena, Claire; Pirrie, Sarah; Southgate, Elizabeth; Talwalkar, Amruta; Teh, William; Turner, Elliot; Wallis, Matthew G; McIntosh, Stuart; Morgan, Jenna; Nabi, Zohal; Paramasivan, Sangeetha; Pinder, Sarah; Potter, Shelley; Roberts, Tracy; Sharma, Nisha; Stobart, Hilary; Taylor-Phillips, Sian; Rea, D

This article was not externally reviewed at BMJ Open. Protocols that have been independently assessed prior to submission to BMJ Open are usually fast-tracked to publication on the grounds that further substantial changes will not be possible. This independent assessment will usually be external, independent review for both a substantial grant award from a non-commercial or government funder and ethics approval.
